# IQGAP2 acts as an independent prognostic factor and is related to immunosuppression in DLBCL

**DOI:** 10.1186/s12885-021-08086-y

**Published:** 2021-05-25

**Authors:** Tianjiao Tang, Jing Wang, Lidan Zhang, Ying Cheng, Laura Saleh, Yanni Gu, Hongbin Zhang

**Affiliations:** 1 Department of Hematology, The First Affiliated Hospital of Chongqing Medical University, No 1, Youyi Road, Yuzhong District, Chongqing, 400016 China; 2 Department of General Practice, University of Chinese Academy of Sciences Chongqing Hospital, Chongqing, China; 3 Department of Neurosurgery, Johns Hopkins University School of Medicine, Baltimore, MD USA; 4 Department of Neuroscience, Johns Hopkins University, Baltimore, MD USA

**Keywords:** DLBCL, IQGAP2, Immunosuppression, Prognostic factor, Independent factor

## Abstract

**Background:**

Almost one-third of patients with diffuse large B-cell lymphoma (DLBCL) cannot be cured with initial therapy and will eventually succumb to the disease. Further elaboration of prognostic markers of DLBCL will provide therapeutic targets. IQ motif-containing GTPase activating protein 2 (IQGAP2) acts as a tumour suppressor in hepatocellular, prostate, and gastric cancers. However, the role of IQGAP2 in DLBCL remains unclear.

**Methods:**

We collected mRNA expression data from 614 samples and the corresponding clinical information. The survival time of patients was compared between groups according to the mRNA expression level of IQGAP2. Survival analyses were performed in different subgroups when considering the effect of age, tumour stage, serum lactate dehydrogenase (LDH) concentration, performance status, and the number of extra nodal disease sites. The biological processes associated with IQGAP2-associated mRNAs were analysed to predict the function of IQGAP2. The correlation of IQGAP2 mRNA with immunosuppressive genes and leukocyte infiltration were analysed.

**Results:**

The overall survival of patients with increased IQGAP2 mRNA levels was reduced even after aggressive treatment independent of age, tumour stage, serum LDH concentration, performance status, and the number of extra nodal disease sites. Furthermore, the biological processes of IQGAP2-associated mRNAs were mainly immune processes. IQGAP2 mRNA expression was correlated with the expression of immunosuppressive genes and leukocyte infiltration.

**Conclusion:**

IQGAP2 mRNA is an independent prognostic factor and is related to immunosuppression in DLBCL. This discovery may provide a promising target for further development of therapy.

**Supplementary Information:**

The online version contains supplementary material available at 10.1186/s12885-021-08086-y.

## Background

Diffuse large B-cell lymphoma (DLBCL) is the most common form of lymphoma and accounts for 25–35% of non-Hodgkin lymphomas [1]. Cyclophosphamide, doxorubicin, vincristine, and prednisone (CHOP) chemotherapy have been the predominant therapies for several decades. In the past two decades, the results of several phase III trials have established a regimen that utilizes a combination of rituximab and CHOP (R-CHOP) to achieve a better result. Therefore, it has become the standard-of-care therapy for patients with DLBCL, with 50–70% of patients typically being cured using this approach [2, 3]. However, the prognoses of the remaining patients who require alternative therapy are poor, and the majority of them will eventually succumb to their disease [4].

The identification of biologically distinct subtypes of DLBCL is a milestone, and gene expression profiling studies have distinguished three molecular subtypes of DLBCL, which are known as germinal centre B-cell-like (GCB) DLBCL, activated B-cell-like (ABC) DLBCL, and unclassified (UC) DLBCL [5, 6] and are correlated with a differential response to therapy. Patients with these DLBCL subtypes have significantly different gene expression profiles and overall survival rates [6–9]. Therefore, we believe that there must be some biological characteristics in DLBCL associated with poor prognosis. Further investigation into the prognostic biomarkers in refractory DLBCL is essential for identifying patients who are unlikely to be cured by R-CHOP and for developing potential therapeutic targets. The development of monoclonal antibody (mAb) therapy directed against CD20 has had the biggest clinical impact on the treatment of DLBCL [10]. This indicates that targeted therapy is promising for treatment of DLBCL. Gene risk scores based on the expression of 6 genes have been proposed to predict the outcome of DLBCL patients [11]. However, no further application or new therapy has been developed that utilizes these genes. Further insight into prognostic biomarkers is urgently needed to develop new therapeutic strategies for refractory DLBCL.

IQ motif-containing GTPase activating protein (IQGAP) 2 is a protein coding gene, and IQGAP2 is a member of the IQGAP family. Mammals express three isoforms of IQGAP: IQGAP1, IQGAP2 and IQGAP3. Their compositions are all similar, and they regulate the cytoskeleton, cytokinesis and carcinogenesis [12]. Despite their 62% sequence identity, IQGAP1 is an oncogene, while IQGAP2 has been reported as a tumour suppressor in hepatocellular, prostate, and gastric carcinomas [13]. However, the role of IQGAP2 in DLBCL remains unclear.

In this study, we explored the mRNA expression of IQGAPs in 1457 cell lines. We also collected a total of 614 samples with mRNA microarray data to identify the relationship between the mRNA expression pattern of IQGAP2 and the clinical outcome. Furthermore, we examined the correlation between IQGAP2 mRNA and immune processes in DLBCL.

## Methods

### Datasets

The GSE10846 and GSE11318 datasets were downloaded from the Gene Expression Omnibus (GEO), including the Series Matrix File and GPL570. GSE11318 is a retrospective dataset including 74 ABC, 71 GCB, 31 PMBL and 27 UC clinical samples, and the survival information of 200 patients was recorded. CHOP-Like Regimen was received by 164 patients of the GSE11318, and the treatment of the other 40 patients remained unknown. GSE10846 is another retrospective dataset including 181 clinical samples from CHOP-treated patients and 233 from R-CHOP-treated patients. GSE10846 includes 167 ABC, 183 GCB, and 64 UC clinical samples, and all of the survival information was recorded. GSE72056 was downloaded from the GEO. Single-cell RNA sequencing (RNA-seq) was applied to 4645 single cells isolated from 19 melanoma patients. All cells were divided into melanoma cells, T cells, B cells, macrophages, endothelial cells, cancer-associated fibroblasts (CAFs), and NK cells.

Data on the expression of mRNA in cancer cell lines was downloaded from the Cancer Cell Line Encyclopedia (CCLE, https://portals.broadinstitute.org/ccle). The results of both microarray and RNA sequencing were analysed. The box plot was sorted and coloured according to the average expression of a mRNA in several cell lines derived from a particular form of cancer.

Data from immunohistochemistry (IHC) staining of lymphoma tissues was downloaded from The Human Protein Atlas. The tissues were stained with the HPA037404 or CAB004241 antibody.

The relevance of IQGAP2 to the survival of hepatocellular and kidney clear cell carcinoma patients was assessed in TIMER (https://cistrome.shinyapps.io/timer/), which is a comprehensive resource for the systematic analysis of diverse cancer types.

### Survival analysis

Survival analyses were conducted with a log-rank (Mantel-Cox) test in GraphPad Prism 8.01. The low and high groups were separated according to the mRNA expression of IQGAP2. *P* < 0.05 was considered statistically significant. Multivariate Cox regression analyses were conducted with the Coxph algorithm.

### IQGAP2 mRNA expression in subtypes

The mean IQGAP2 mRNA expression values were compared in ABC, GCB, UC and PMBL. The unpaired t-test was used to compare the means of two groups, and a two-tailed *p* value of < 0.05 was considered statistically significant.

### Biological process (BP) enrichment analysis

Pearson correlation coefficients (r values) between IQGAP2 and all other genes were calculated in RStudio 1.1.453. IQGAP2-associated genes were defined as genes for which r > 0.4. BP analysis of IQGAP2-associated genes was conducted in The Database for Annotation, Visualization and Integrated Discovery (DAVID) v6.8. Column-folded line graphs were generated in Excel 2016.

### Heatmaps of IQGAP2 mRNA and mRNAs of immune-related genes

The mRNA expression files were uploaded to Morpheus (https://software.broadinstitute.org/morpheus/). The Z score (subtract mean, divide by standard deviation) of all of the expression data was calculated and is shown via a heatmap. All of the samples were sorted according to the IQGAP2 expression. Heatmaps were downloaded from Morpheus.

### Leukocyte infiltration

The Estimate of Stromal and Immune Cells in Malignant Tumors from Expression Data (ESTIMATE) package was developed by Yoshihara et al. to estimate the number of infiltrating nontumour cells and tumour purity [14]. Cell-type Identification By Estimating Relative Subsets Of RNA Transcripts (CIBERSORT, https://cibersort.stanford.edu/), also known as in silico flow cytometry [15], was used to evaluate the percentage of leukocyte infiltration. Both the ESTIMATE and CIBERSORT results were uploaded to Morpheus. All samples were sorted according to their IQGAP2 expression.

## Results

### IQGAP2 mRNA is predominantly expressed in malignant haematologic cells

**Fig. 1:**
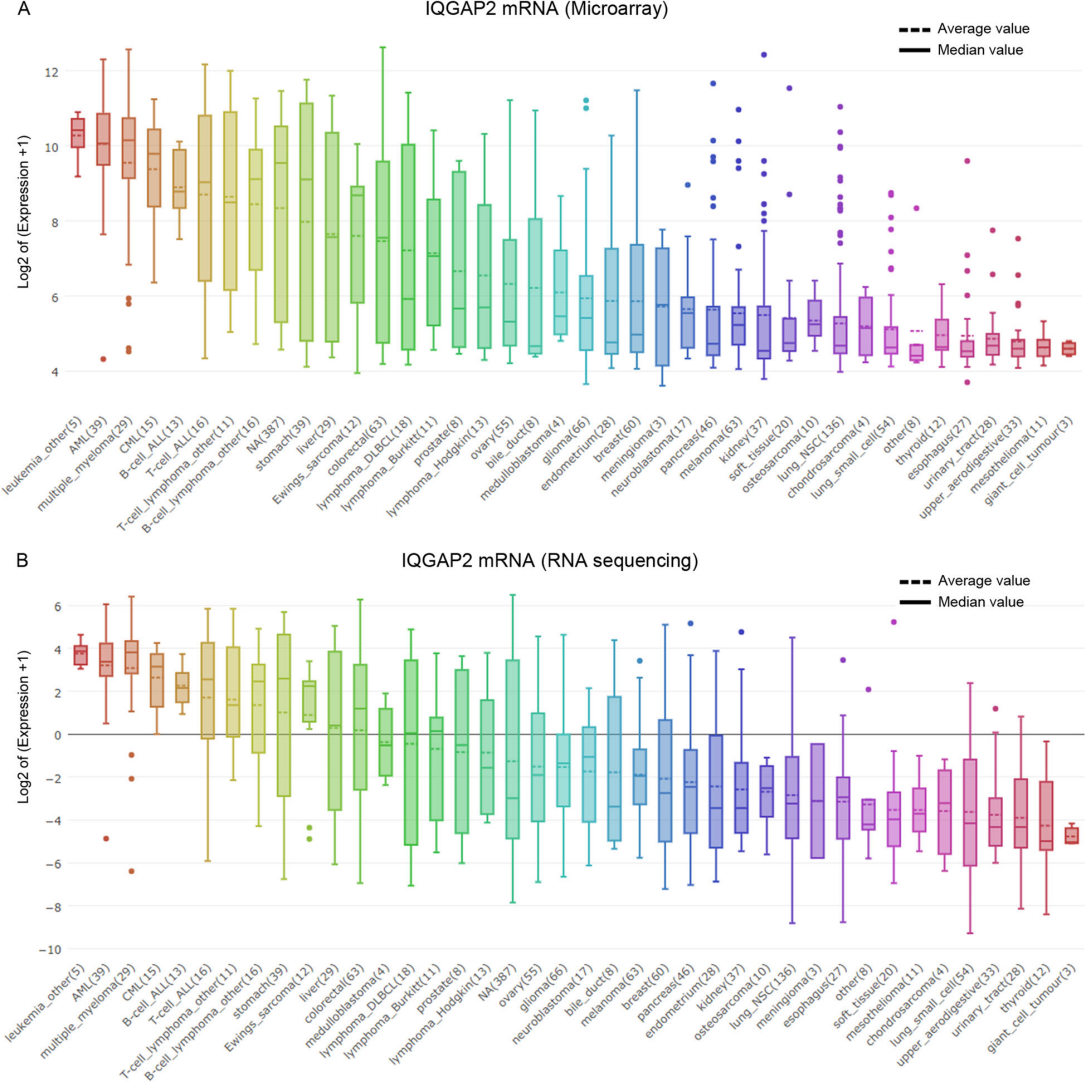
IQGAP2 mRNA in cancer cell lineages. IQGAP2 mRNA in cancer cell lineages detected by microarray (**a**) and RNA sequencing (**b**). The box plots are sorted by the average value of mRNA expression

The expression pattern of IQGAP mRNA in various cancer cell lines in CCLE was examined. First, the results showed that IQGAP2 mRNA is increased in liver cancer cell lines compared to that in glioma, lung, and kidney cancer cell lines (Fig. 1. A and B), which is consistent with previous reports [16]. Second, both the microarray and RNA sequencing results showed that IQGAP2 mRNA is predominantly expressed in malignant haematologic cells (Fig. 1. A and B). However, cells in haematologic malignancies exhibit relatively low expression of IQGAP1 mRNA and IQGAP3 mRNA when compared to other cancer cells (Supplementary Fig. 1. A-D).

In addition, the single-cell sequencing results of melanoma showed that T cells, macrophages, and NK cells expressed significantly more IQGAP2 than melanoma cells (Supplementary Fig. 2. A, B).

### IQGAP2 mRNA expression is inversely related to DLBCL survival

**Fig. 2:**
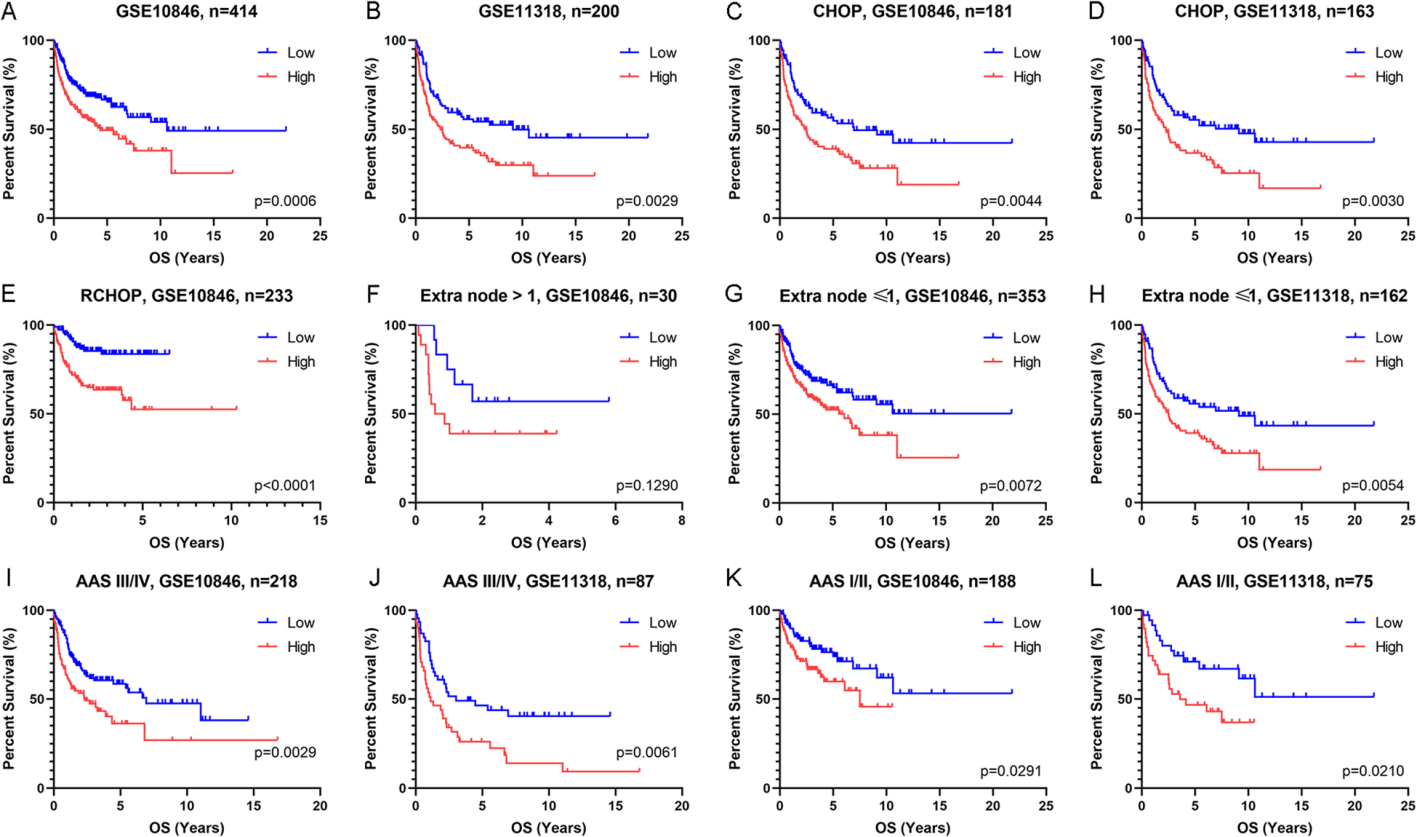
Survival analyses adjusted according to therapy, extranodal sites and stages. Patients with DLBCL were separated into two groups according to the mRNA expression of IQGAP2 in every figure. Survival analyses of all patients in the GSE10846 (**a**) and GSE11318 (**b**) datasets. Survival analyses of patients treated with CHOP (**c** and **d**) or R-CHOP (**e**). Survival analyses of patients with extranodal sites > 1 (**f**) or ≤ 1 (**g**, **h**). Survival analyses of patients with Ann Arbor stage III/IV (**i** and **j**) or I/II (**k** and **l**). AAS, Ann Arbor stage

The survival analyses showed that patients with high IQGAP2 mRNA exhibited shorter survival times than those with low IQGAP2 mRNA in both the GSE10846 and GSE11318 datasets (Fig. 2. A and B). Considering the therapeutic effect on survival, we conducted a survival analysis in patients with the same chemotherapy regimens. We found that the patients with high IQGAP2 mRNA levels still lived for a shorter amount of time when treated with CHOP and R-CHOP (Fig. 2. C-E). Based on the TCGA data, patients with hepatocellular and kidney cancers with high IQGAP2 mRNA levels live for a longer time (Supplementary Fig. 3. A, B).

**Fig. 3:**
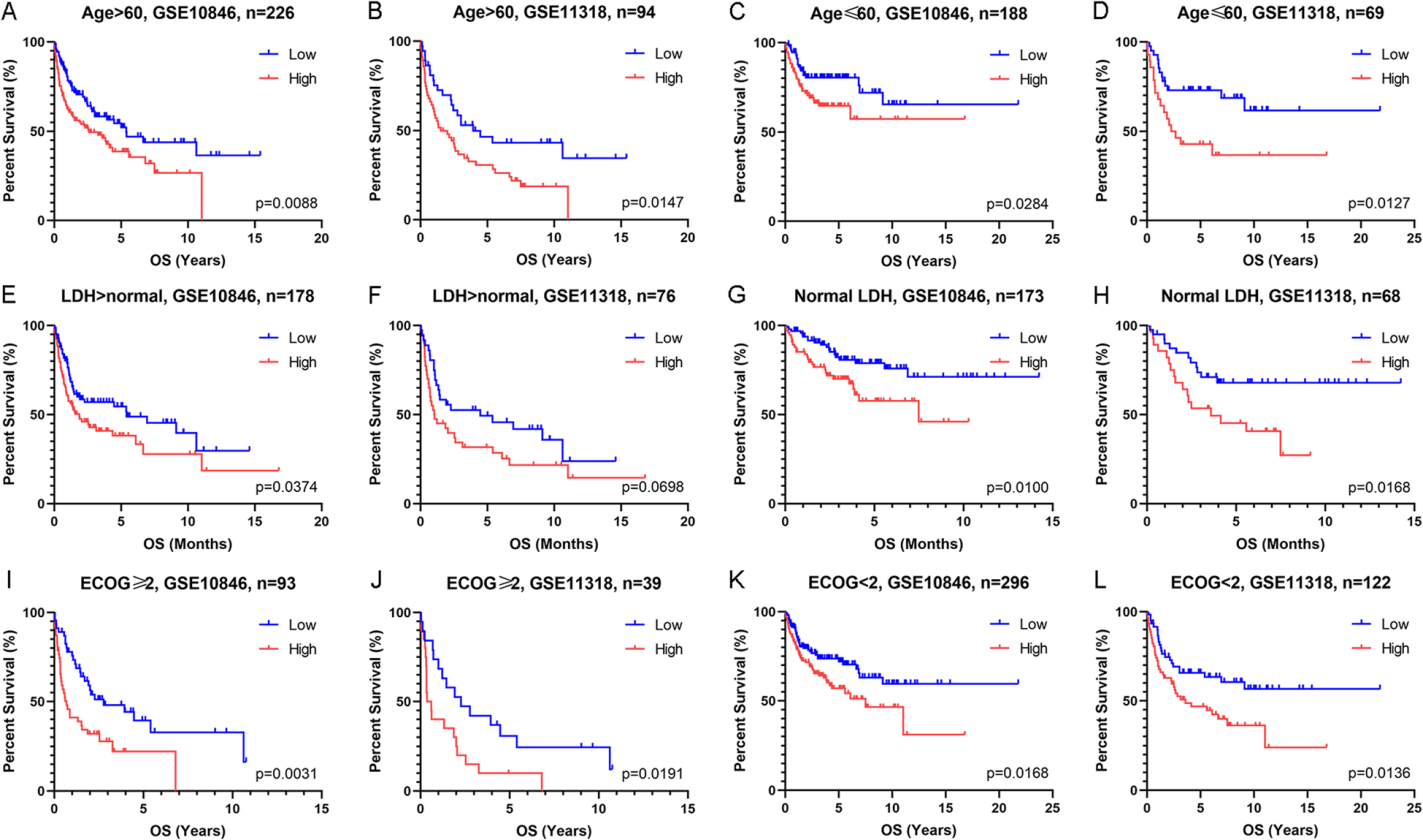
Survival analyses adjusted by age, LDH and ECOG. Patients with DLBCL were separated into two groups according to the mRNA expression of IQGAP2 in every figure. Survival analyses of patients with age > 60 (**a** and **b**) or ≤ 60 (**c** and **d**). Survival analyses of patients with LDH > normal (E and F) or normal LDH (G and H). Survival analyses of patients with ECOG ≥2 (I and J) or <2 (K and L). LDH, serum lactate dehydrogenase concentration. ECOG, performance status of the Eastern Cooperative Oncology Group

**Fig. 4:**
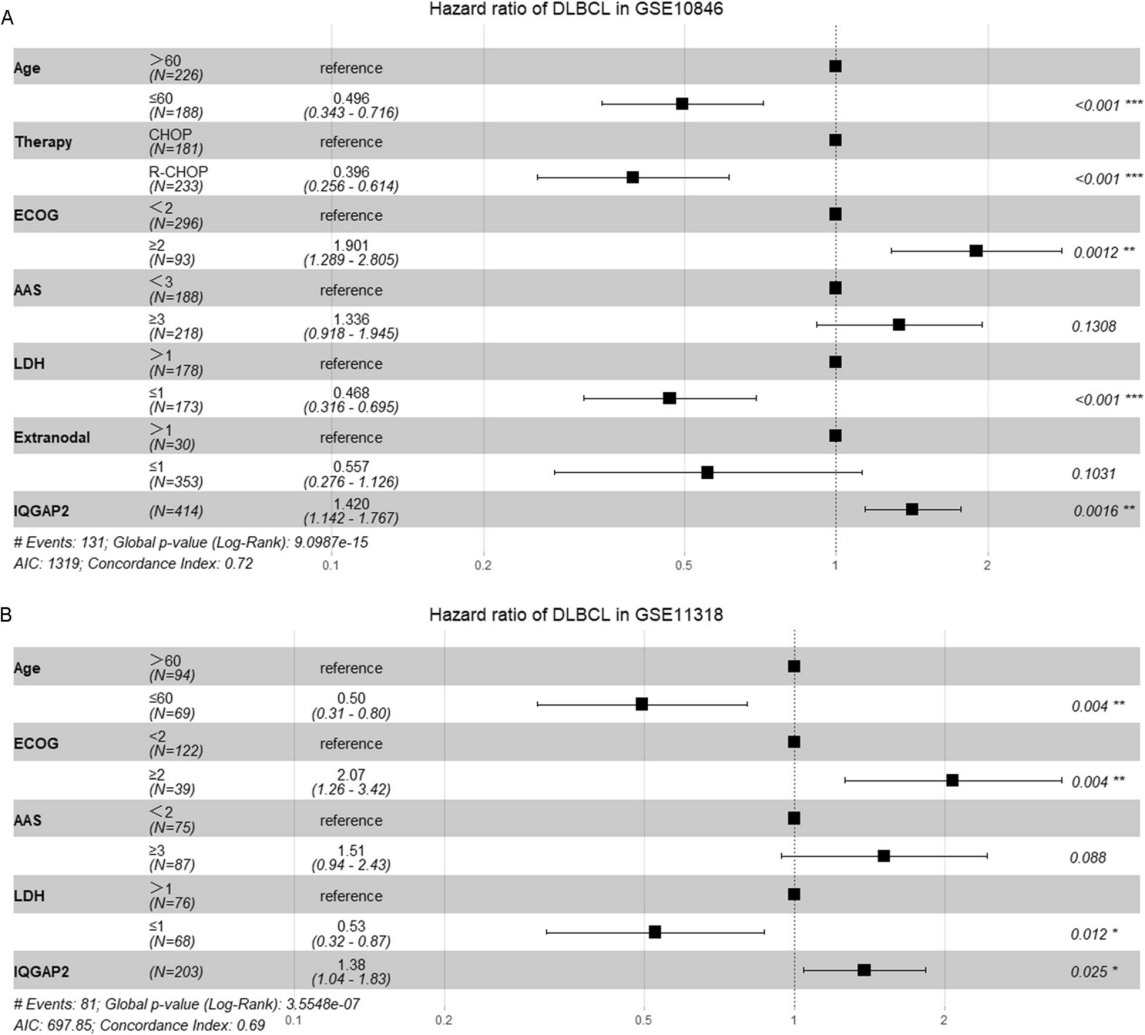
Multivariate Cox regression analyses for survival in DLBCL. The potential prognostic factors were analysed by multivariate Cox regression in GSE10846 (**a**) and GSE11318 (**b**). The results are shown in forest plots

The international prognostic index is a well-accepted system to predict the outcome of DLBCL. It includes age, Ann Arbor stage (AAS), serum lactate dehydrogenase (LDH) concentration, performance status according to the Eastern Cooperative Oncology Group (ECOG), and number of extra nodal disease sites. Considering the impact of these confounding factors on survival, we conducted survival analyses in subgroups of patients (Fig. 2. F-L and Fig. 3. A-L). The survival time of patients with high IQGAP2 mRNA levels was evidently shorter. In addition, we conducted multivariate Cox regression analyses to evaluate the prognostic value of IQGAP2 expression and other prognostic factors. The results showed that the expression of IQGAP2 along with age, therapy, ECOG, and LDH were prognostic indicators in GSE10846 (Fig. 4.A). In GSE11318, the expression of IQGAP2 was still correlated with survival after adjusting for other factors, including age, ECOG, AAS, and LDH (Fig. 4.B).

### IQGAP2 expression in subgroups of DLBCL

**Fig. 5:**
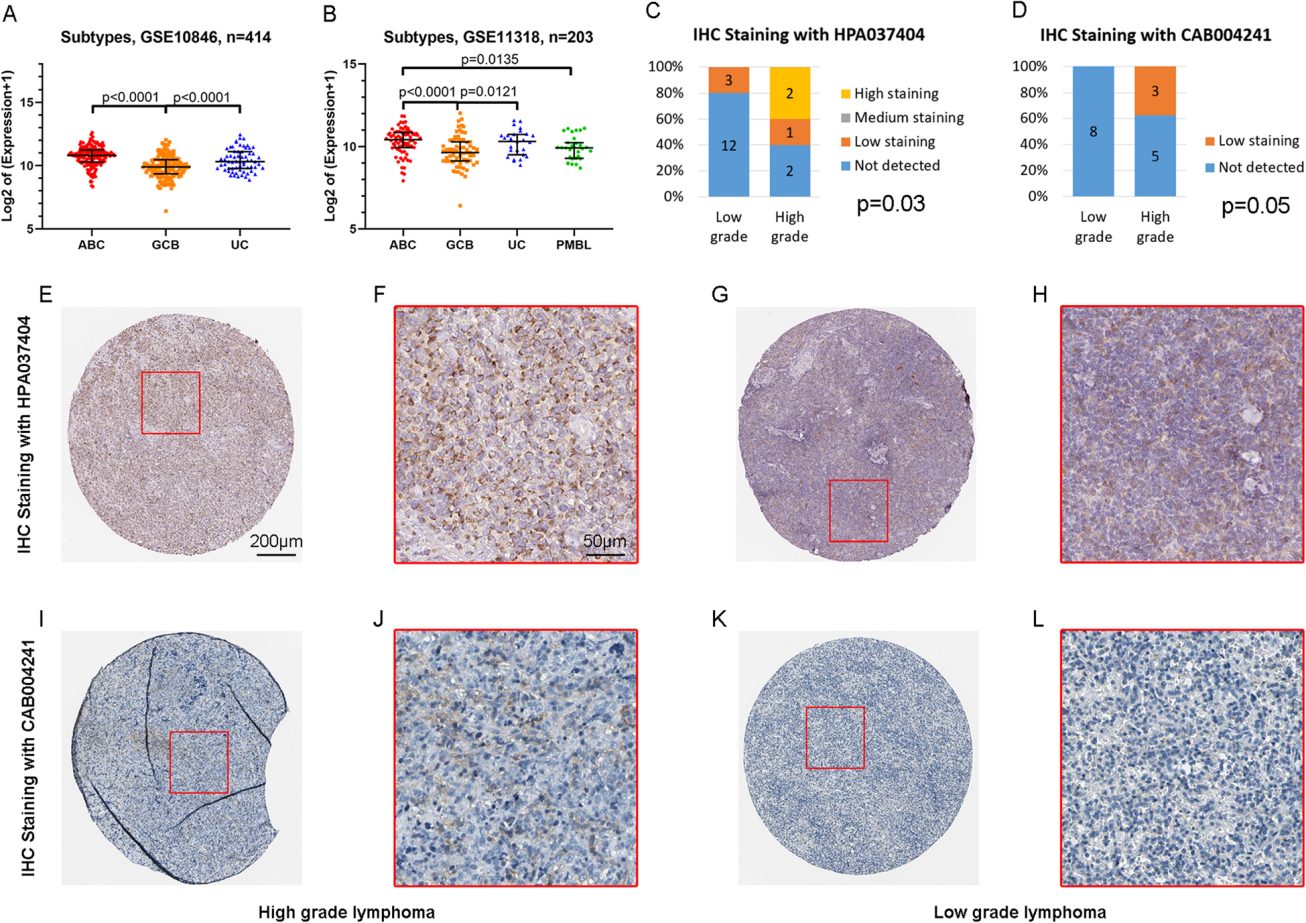
IQGAP2 mRNA distribution in DLBCL subtypes. **a** IQGAP2 mRNA expression in ABC, GCB and UC DLBCL tissues from GSE10846. **b** IQGAP2 mRNA expression in ABC, GCB, UC and PMBL DLBCL tissues from GSE11318. GCB, germinal centre B-cell-like. ABC, activated B-cell-like. UC, unclassified. PMBL, primary mediastinal B-cell lymphoma. C-L. The IHC staining of IQGAP2 protein in lymphoma tissues was compared in low grade and high grade with HPA037404 (**c**, **e-h**) or CAB004241 (**d**, **i-l**) antibody

Patients with GCB DLBCL exhibited longer survival rates than those with non-GCB DLBCL (including ABC and UC) [7, 9]. Our results show that samples from GCB DLBCL express lower IQGAP2 mRNA than ABC DLBCL (Fig. 5. A and B). This indicates that IQGAP2 mRNA is related to the malignancy of DLBCL. At the protein level, high-grade lymphoma expressed more IQGAP2 than low-grade lymphoma (Fig. 5. C-L).

### IQGAP2 is associated with immunosuppression

**Fig. 6:**
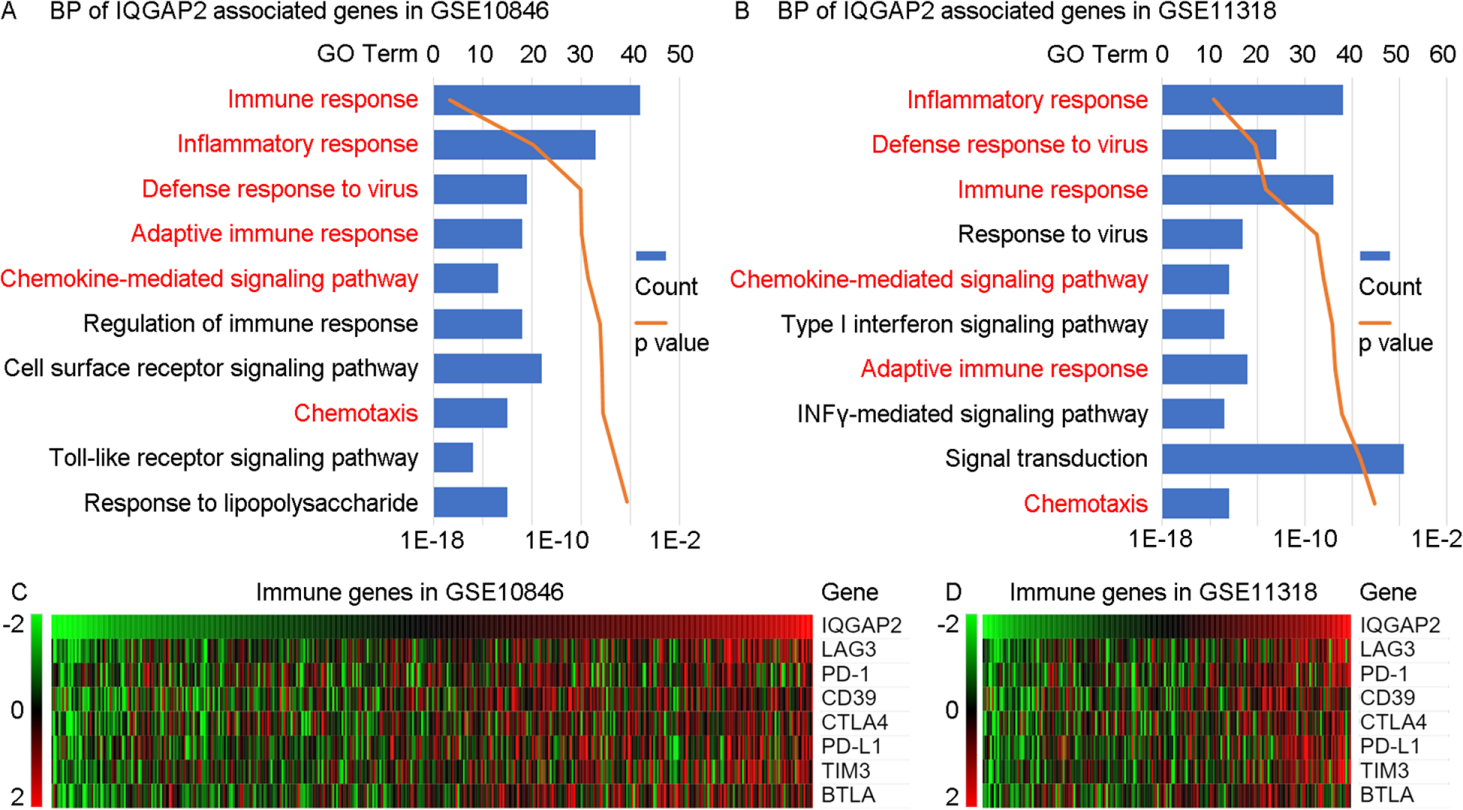
The association between IQGAP2 mRNA and immunosuppression in DLBCL. The biological process enrichment analyses of GSE10846 (**a**) and GSE11318 (**b**) show that IQGAP2-associated mRNAs are mainly concentrated in immune processes, inflammatory response, defence response to virus, adaptive immune response, chemokine-mediated signalling pathway and chemotaxis. The blue column shows the gene counts, and the orange curve shows the *p* value. The correlation between IQGAP2 mRNA expression and the expression pattern of immunosuppressive mRNA was examined in GSE10846 (**c**) and GSE11318 (**d**). The samples were sorted by IQGAP2 mRNA expression

Pearson correlation coefficients (r values) between IQGAP2 mRNA expression and every other type of mRNA expression were calculated. The mRNAs with an r value > 0.4 are defined as IQGAP2-associated genes. All of these IQGAP2-associated genes were uploaded to DAVID to conduct enrichment analyses. The results of biological progress show that IQGAP2-associated mRNA is mainly associated with immune processes (immune response, inflammatory response, defence response to virus and adaptive immune response) and chemotaxis (chemokine-mediated signalling pathway) (Fig. 6. A, B).

Therefore, immune processes may promote or inhibit cancer development. We further examined the expression correlation between IQGAP2 and immunosuppressive genes (LAG3, PD-1, CD39, CTLA4, PD-L1, TIM3, BTLA) [17–19]. The results show that IQGAP2 mRNA expression is positively correlated with these immunosuppressive genes (Fig. 6. C, D). This indicates that IQGAP2 might be involved in the immunosuppression in DLBCL.

### IQGAP2 is associated with leukocyte infiltration

**Fig. 7:**
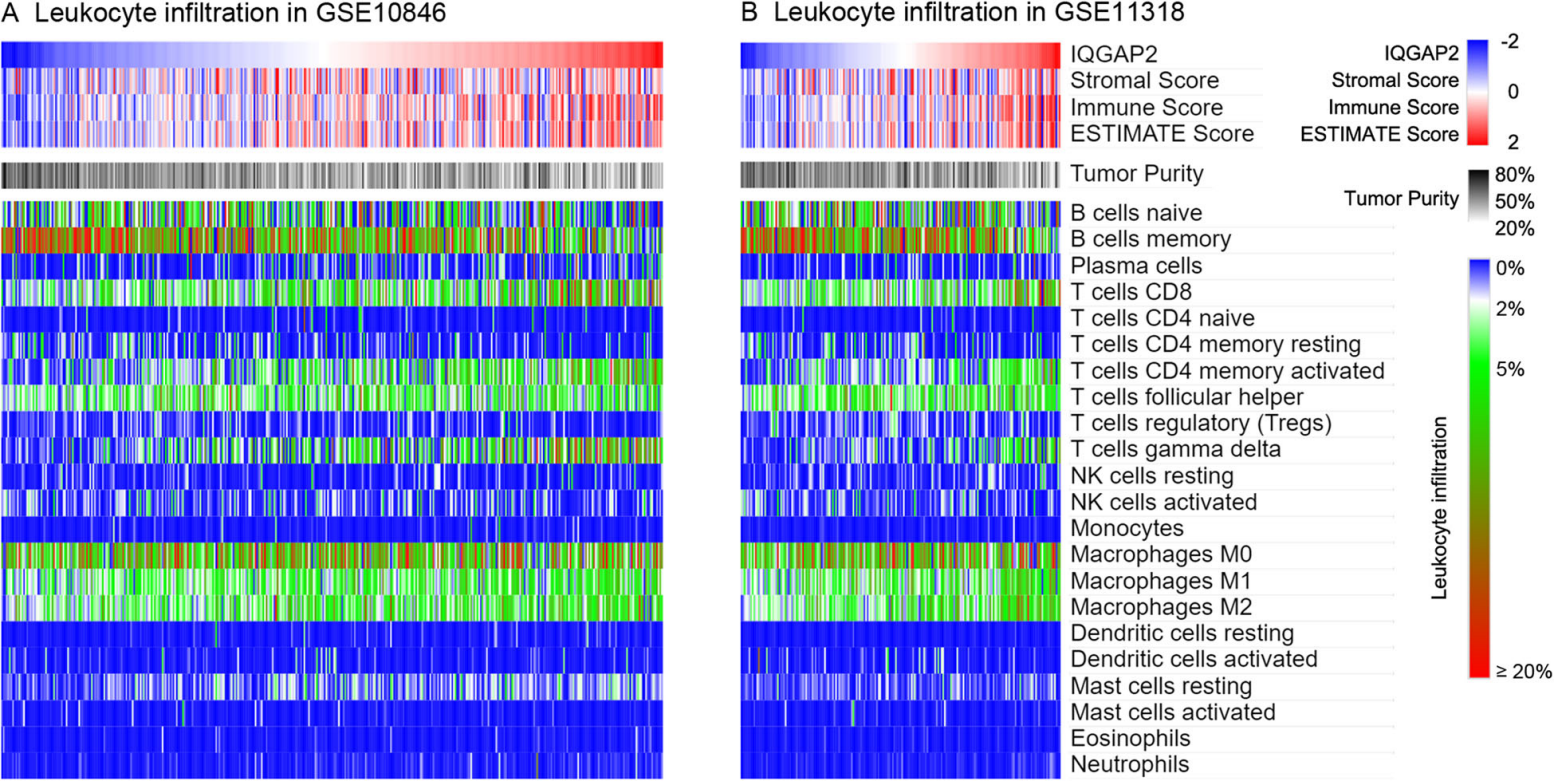
The association between IQGAP2 mRNA and leukocyte infiltration. The association between IQGAP2 mRNA and tumour purity and leukocyte infiltration are conducted in GSE10846 (**a**) and GSE11318 (**b**). The stromal score, immune score, ESTIMATE score and tumour purity were calculated by the ESTIMATE algorithm. The samples were sorted by IQGAP2 mRNA expression

The ESTIMATE algorithm was used to calculate the tumour purity. CIBERSORT, a new method called “in silico flow cytometry,” was then used to further assess the leukocyte populations in the tumour microenvironment of DLBCL. The results indicated that IQGAP2 mRNA is negatively associated with tumour purity and positively correlated with the immune score (Fig. 7. A, B), which suggests that IQGAP2 plays an important role in the immune microenvironment. In addition, we found that IQGAP2 exhibited a positive correlation with CD8 T cells, CD4 memory-activated T cells, and gamma delta T cell infiltration (Fig. 7. A, B). IQGAP2 also showed a negative correlation with memory B cell populations (Fig. 7. A, B).

## Discussion

It is widely accepted that IQGAP2 is predominantly expressed in the liver rather than in the heart, brain, spleen, lung, kidney, testis, and skeletal muscle. This result was detected by an RNA blot in 1996 [16]. In the last decade, IQGAP2 was reported to be found in cancer tissues of prostate [20], lung, breast, liver, kidney, and colorectal cancer [21]. IQGAP2 is also expressed in podocytes [22] and glomerular endothelial cells [23]. However, these studies ignored the expression of IQGAP2 in leukocytes. Our study shows that IQGAP2 mRNA levels are increased in liver cancer cell lines compared to those in glioma, lung, and kidney cancer cell lines (Fig. 1), which is consistent with previous reports. Despite this, we found that cell lines derived from haematologic malignancies exhibit higher IQGAP2 levels than liver cancer cell lines (Fig. 1), which does not occur for IQGAP1 and IQGAP3 (Supplementary Fig. 1). Furthermore, single-cell sequencing showed that IQGAP2 is predominantly expressed on leukocytes but not melanoma cells (Supplementary Fig. 1). It has been reported that mice lacking IQGAP2 are resistant to chemically induced colitis and experience diminished neutrophil and macrophage production and recruitment [24]. This shows that IQGAP2 is predominantly expressed in leukocytes but not in the liver. More work is needed to confirm the expression pattern of IQGAP2 in haematologic cells.

IQGAP2 is a well-known tumour suppressor. IQGAP2 is decreased in several cancers, including hepatocellular [25], prostate [20], and gastric [26] carcinomas. Of note, IQGAP2 deficiency results in hepatocellular carcinoma in mice, but IQGAP1 and IQGAP2 double disrupted mice have less hepatocellular carcinoma and normal survival. This suggests that IQGAP1 and IQGAP2 have inverse functions [27]. Our results verify that IQGAP2 mRNA expression is correlated with the overall survival of patients with hepatocellular and kidney carcinoma (Supplementary Fig. 3). In contrast, we found that IQGAP2 mRNA expression is inversely related to the survival of patients with DLBCL (Fig. 2 and Fig. 3). Given that IQGAP2 mRNA is also related to immunosuppression and leukocyte infiltration (Fig. 6 and Fig. 7), we propose that the high expression of IQGAP2 in leukocytes and various leukocytic components in DLBCL may contribute to the immunosuppressive role of IQGAP2 in DLBCL.

Immune evasion plays a key role in DLBCL oncogenesis. The development of DLBCL requires both deregulated expression of the oncogene as well as escape from T cell-mediated tumour surveillance [28]. Most DLBCL cells fail to express both β2-microglobulin and CD58, which are required for immune recognition of malignant cells [29]. The expression of PD-L1 is related to poor outcomes [30]. In addition, nontumour cell infiltration into DLBCL also leads to a poor prognosis [31]. When the tumour immune microenvironment was impaired by high PD-1 expression on CD8+ T cells or PD-L1 expression on T cells and macrophages, patients had significantly worse outcomes after R-CHOP therapy [32]. Immunotherapy provides additional choices for patients with DLBCL that is refractory to chemotherapies or that has relapsed after stem cell transplantation. The chimeric antigen receptor T-cell (CAR-T) therapeutic tisagenlecleucel produced a 52% overall response rate in these patients (40% of the patients had complete responses, and 12% had partial responses) [33]. Although nivolumab (anti-PD-1) monotherapy showed a low overall response rate among these patients, two patients survived for 30 and 38 months after the first treatment [34]. Our data show that IQGAP2-associated genes are associated mostly with immune processes (Fig. 6. A and B) and that IQGAP2 mRNA is correlated with immunosuppressive genes (Fig. 6. C and D). Samples with relatively higher IQGAP mRNA levels consistently exhibited higher infiltration of leukocytes (Fig. 7). These results indicate that IQGAP2 might be involved in immunosuppression in DLBCL.

IQGAP2 is mainly proposed as a cell motility modulator. IQGAP2 recruits active Rac1 to promote membrane ruffling by interacting with AKAP220 [35]. IQGAP2 regulates actin assembly downstream of thrombin stimulation by interacting with Arp2/3 and F-actin [36]. IQGAP2 also inhibits GTPase activity by interacting with Cdc42 and Rac1 [16]. In addition, IQGAP2 is required for the glomerular filtration barrier by maintaining podocyte structure and function [23]. Moreover, IQGAP2 is an IFNα antiviral effector gene acting nonconventionally through the NF-κB pathway in HCV-infected hepatocytes [37]. Our results show that IQGAP2-associated genes are related to chemotaxis. The effect of IQGAP2 on cell motility in DLBCL needs further investigation.

A previous study showed that IQGAP2 is involved in regulating IFN-stimulated gene (ISG, with known antiviral properties) induction by IFN in an NF-κB-dependent manner in hepatic cells [37]. This indicates that IQGAP2 may be necessary for the activation of the NF-κB pathway. NF-κB is a key oncogenic pathway in DLBCL [38–40]. The activation of the NF-κB pathway dependent on IQGAP2 is probably the main reason for immunosuppression in DLBCL because of its induction of immune checkpoint molecules such as PD-L1 [41]. This speculation is worth further verification.

Our work indicated that IQGAP2 is an independent prognostic factor and is related to immunosuppression in DLBCL. This indication was mainly derived from the results of silico microarray and RNA sequencing. The protein expression of IQGAP2 was compared among different subtypes of DLBCL (Fig. 5). But the prognostic value and immunosuppressive role of IQGAP2 protein in DLBCL need to be further validated in fresh specimens.

## Conclusion

In conclusion, our data show that IQGAP2 mRNA is more highly expressed in several cell lines derived from haematologic malignancies compared to most other cancer cell lines. We also demonstrate that IQGAP2 is an inversely survival-related marker in DLBCL, which differs from its role in other tumours. Furthermore, we propose that IQGAP2 may play a role in immunosuppression in DLBCL. Our work provides evidence for further investigation of IQGAP2 in DLBCL.

## Supplementary Information

**Additional file 1: Figure S1.** IQGAP1 and IQGAP3 mRNA in cancer cell lineages. **Figure S2.** IQGAP2 expression in single cells. **Figure S3.** Survival analyses of IQGAP2 mRNA in hepatocellular carcinoma and kidney clear cell carcinoma.

## Data Availability

The public datasets analyzed during the current study are available in the following repositories: GSE10846 and GSE11318 (https://www.ncbi.nlm.nih.gov/geo/), CCLE (https://portals.broadinstitute.org/ccle/data). Other data that were not relevant to the results presented here are available from the corresponding author upon reasonable request.

## Abbreviations

DLBCL: Diffuse large B-cell lymphoma
IQGAP2: IQ Motif Containing GTPase Activating Protein 2
LDH: Lactate dehydrogenase
CHOP: Cyclophosphamide, doxorubicin, vincristine, and prednisone
R-CHOP: Rituximab, cyclophosphamide, doxorubicin, vincristine, and prednisone
GCB: Germinal center B-cell-like
ABC: Activated B-cell-like
UC: Unclassified
PMBL: Primary mediastinal B-cell lymphoma
mAb: Monoclonal antibody
GEO: Gene Expression Omnibus
CCLE: Cancer Cell Line Encyclopedia
BP: Biological process
DAVID: The Database for Annotation, Visualization and Integrated Discovery
ESTIMATE: The Estimation of Stromal and Immune cells in Malignant Tumors using Expression data
CIBERSORT: Cell-type Identification by Estimating Relative Subsets Of RNA Transcripts
AAS: Ann Arbor stage
ECOG: Eastern Cooperative Oncology Group
