# Supplementary material for: IQGAP2 acts as an independent prognostic factor and is related to immunosuppression in DLBCL
Source: BMC Cancer. 2021 May 25;21:603. doi: 10.1186/s12885-021-08086-y (PMC8152057; doi:10.1186/s12885-021-08086-y)
Supplement: Supplementary file 1 — Additional file 1: Figure S1. IQGAP1 and IQGAP3 mRNA in cancer cell lineages. Figure S2. IQGAP2 expression in single cells. Figure S3. Survival analyses of IQGAP2 mRNA in hepatocellular carcinoma and kidney clear cell carcinoma. [file 12885_2021_8086_MOESM1_ESM.docx]

Supplementary figures


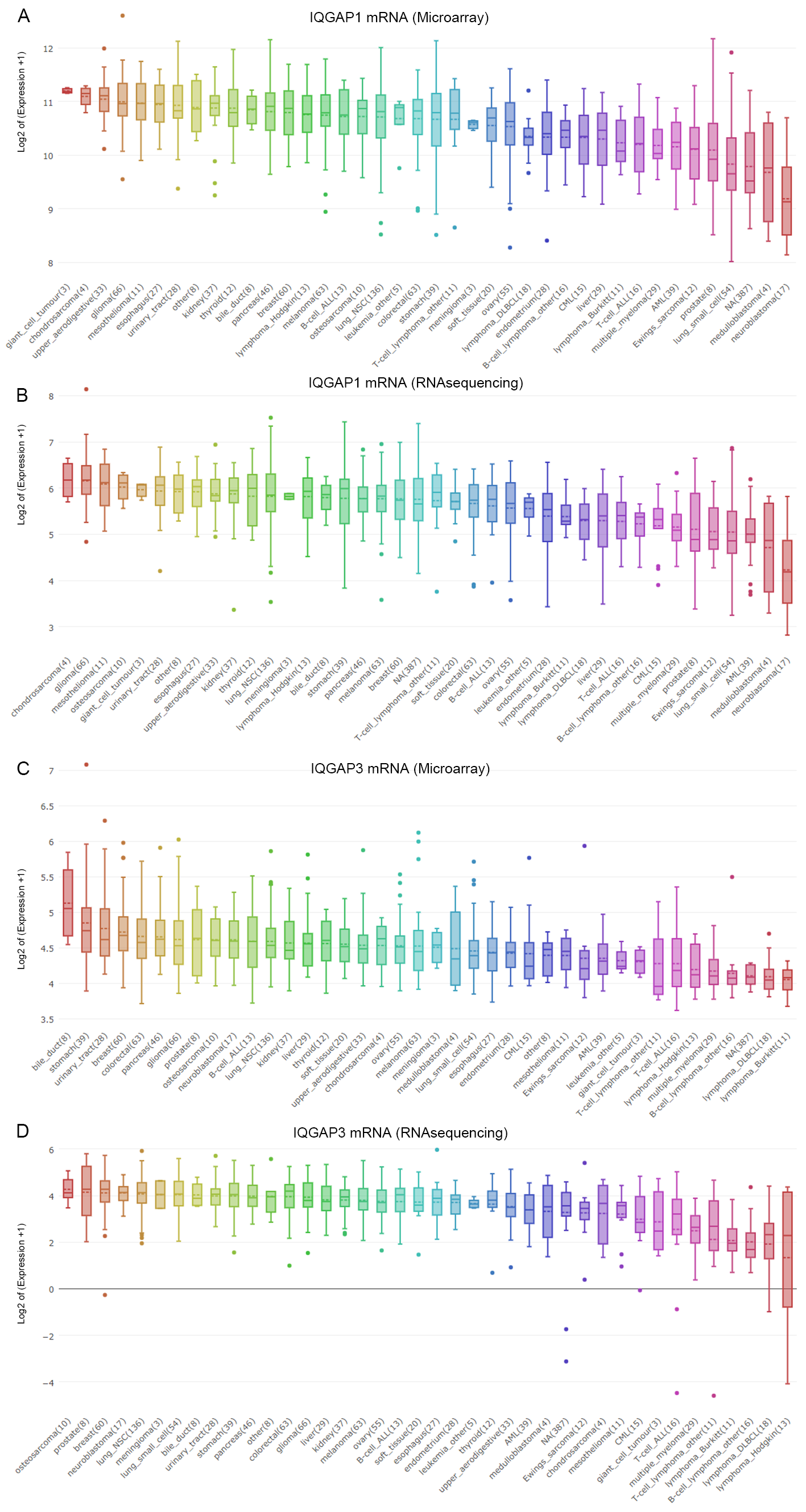


Supplementary Figure 1. IQGAP1 and IQGAP3 mRNA in cancer cell lineages. IQGAP1 mRNA in cancer cell lineages detected by microarray (A) and RNA sequencing (B). IQGAP3 mRNA in cancer cell lineages detected by microarray (C) and RNA sequencing (D). Box plot are sorted by average values.


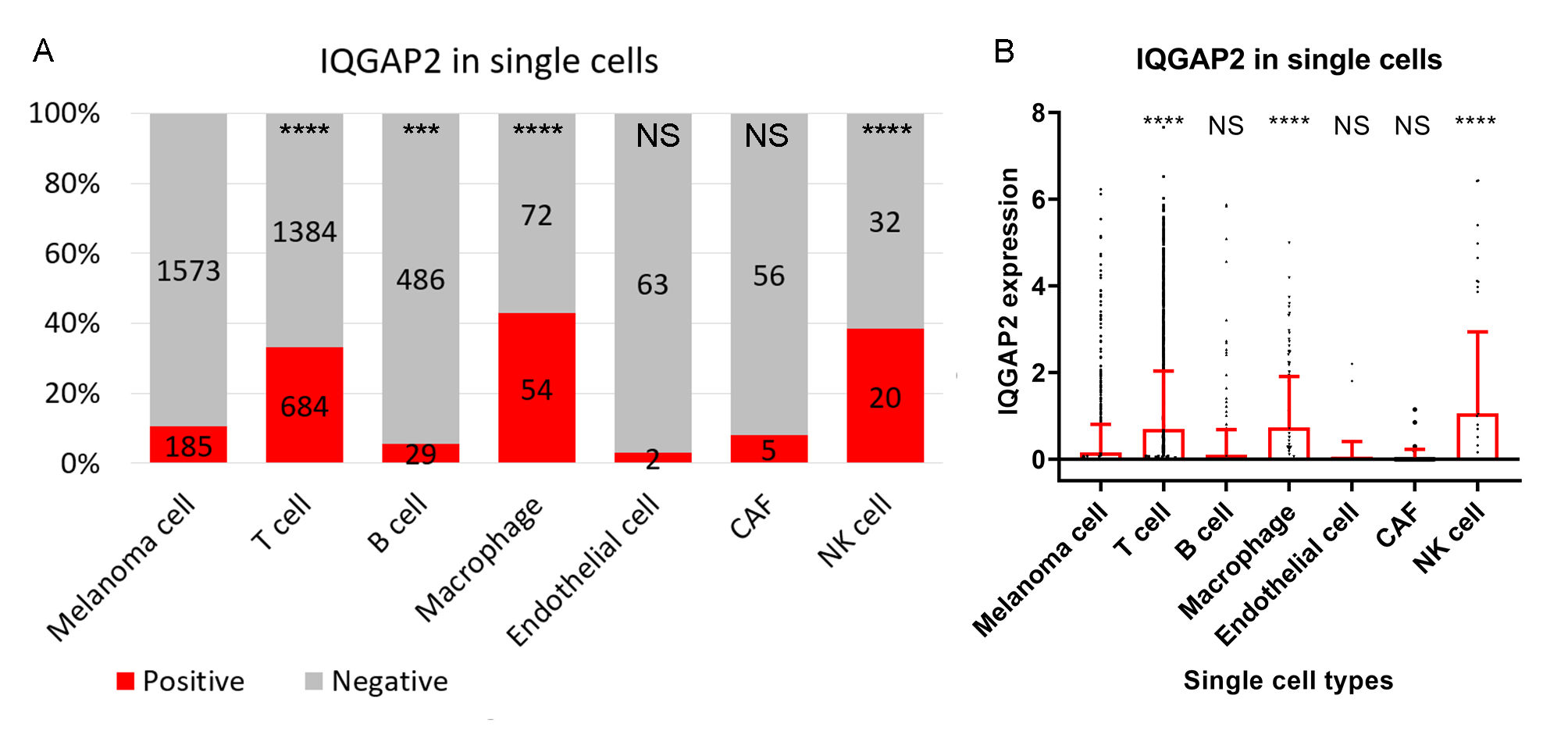


Supplementary Figure 2. IQGAP2 expression in single cells. IQGAP2 mRNA positive ratio (A) and mRNA expression (B) in melanoma cells, T cells, B cells, macrophages, endothelial cells, cancer associated fibroblasts (CAF), and NK cells. ****, p<0.0001. ***, p<0.001. NS, not significant.


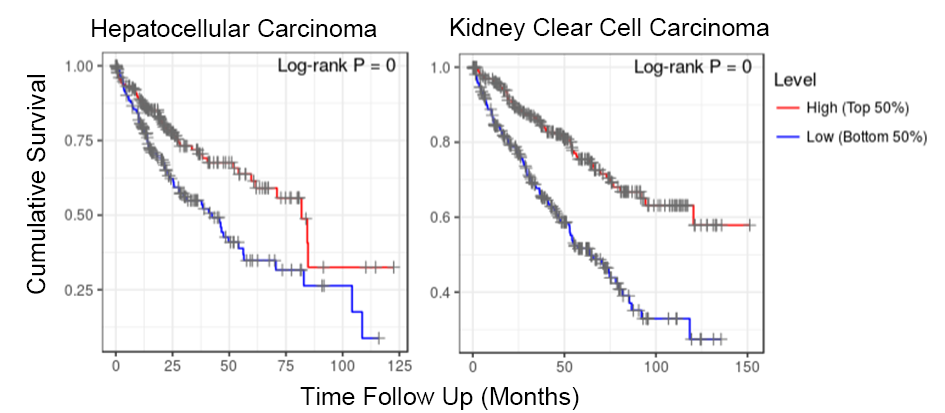


Supplementary Figure 3. Survival analyses of IQGAP2 mRNA in hepatocellular carcinoma and kidney clear cell carcinoma. Patients with hepatocellular carcinoma or kidney clear cell carcinoma are separated into groups according to the expression of IQGAP2 mRNA from tumor tissues.
